# Cataract surgical rates

**Published:** 2018-02-08

**Cataract remains the largest cause of blindness worldwide. Cataract surgical rate, or CSR, measures how many cataract operations are performed per million population in a given year. The map shows the latest available data taken from the IAPB atlas.**

**Figure:**
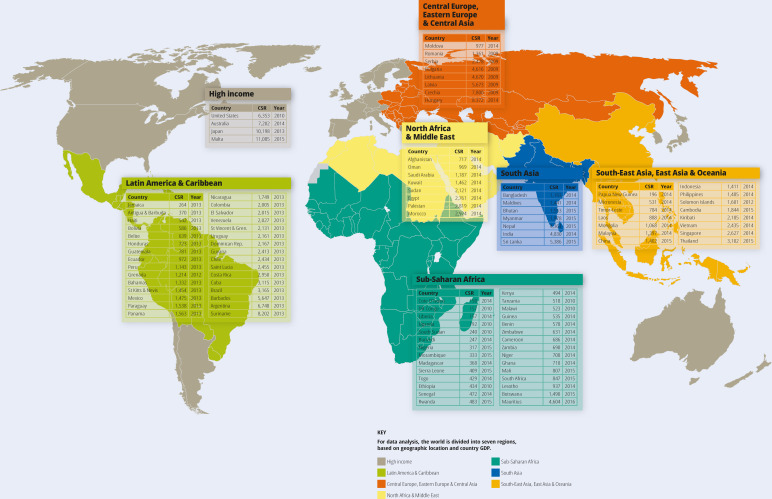


Cataract may cause moderate vision impairment (<6/18 to 6/60), severe vision impairment, (<6/30 to 3/60) or blindness (<3/60). People may have bilateral or unilateral cataract, so it is more useful to consider the number of eyes than the number of people who require surgery.

**Cataract surgical rate (CSR)** is the number of cataract operations performed in one year, per million population. It is a measure of the quantity of cataract services.

CSR needed in each country (the target CSR) is determined by the number of eyes that will develop cataract in one year (the **incidence**).

Incidence is affected by the age structure of a population. Older populations have a higher incidence of cataract than younger populations.

If the number of new cases (the incidence) is higher than the cataract surgical rate, then the backlog (the number of eyes that require cataract surgery), will also be high.

**Figure 1:**
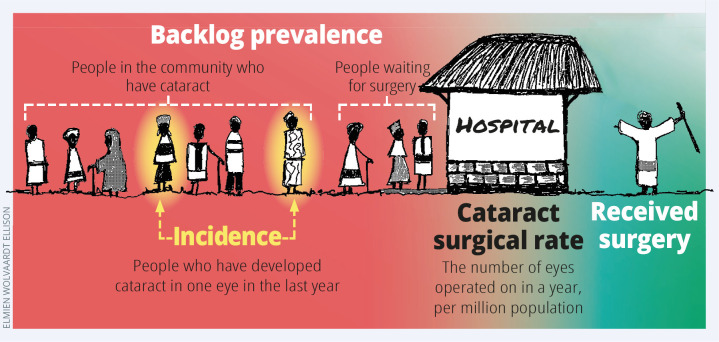
Understanding incidence, backlog and cataract surgical rate

**Figure:**
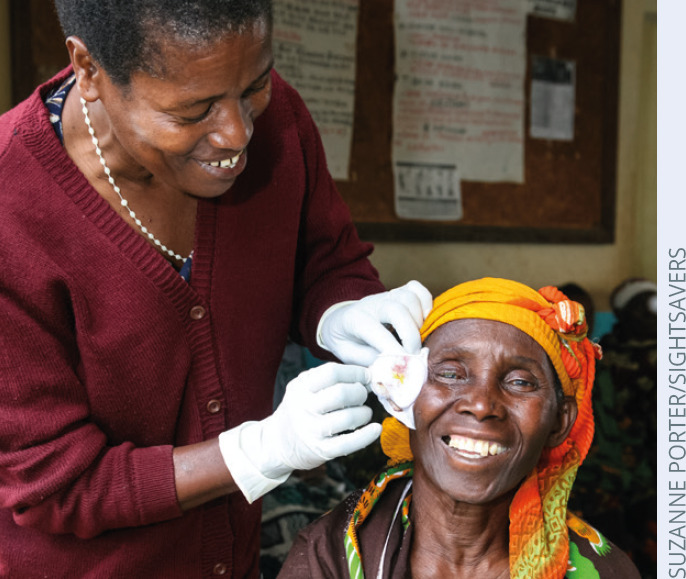
A woman sees for the first time after cataract surgery. TANZANIA

